# Supplementary material for: Ultra-low extracorporeal volume microfluidic leukapheresis is safe and effective in a rat model
Source: Nat Commun. 2025 Feb 24;16:1930. doi: 10.1038/s41467-025-57003-5 (PMC11850925; doi:10.1038/s41467-025-57003-5)
Supplement: Supplementary file 1 — Supplementary information [file 41467_2025_57003_MOESM1_ESM.pdf]

Supplementary Information

Ultra-low extracorporeal volume microfluidic leukapheresis is safe and effective in a rat model

Mubasher Iqbal, Alexandra L. McLennan, Anton Mukhamedshin, Mai T.P. Dinh, Qisheng Liu, Jacob J. Junco, Arvind Mohan, Poyyapakkam R. Srivaths, Karen R. Rabin, Thomas P. Fogarty, III, Sean C. Gifford, Sergey S. Shevkoplyas*, Fong W. Lam*

*Corresponding authors

This document contains:

1. Supplementary Methods
2. Supplementary Note
3. Supplementary Tables 1-3
4. Supplementary Figures 1-7
5. Supplementary References

Supplementary Methods: Imaging Flow Cytometry Analysis

All

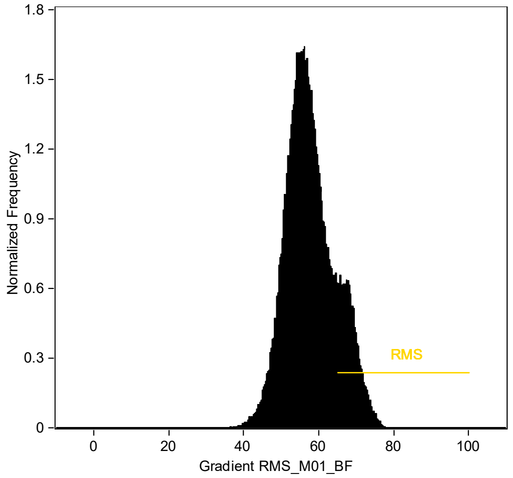

Step 1: Identify cells in focus by using the brightfield channel (CH01; 40X magnification). Cells that are most in focus have a high Gradient RMS (65 to 100).

RMS

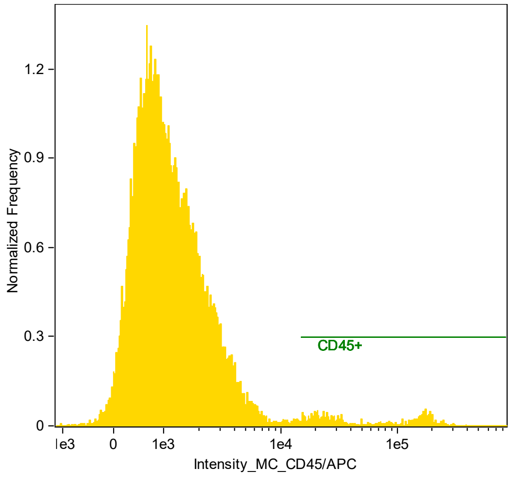

Step 2: Use the RMS population to identify CD45$^+$ (WBC; intensity>15,000) cells.

CD45+

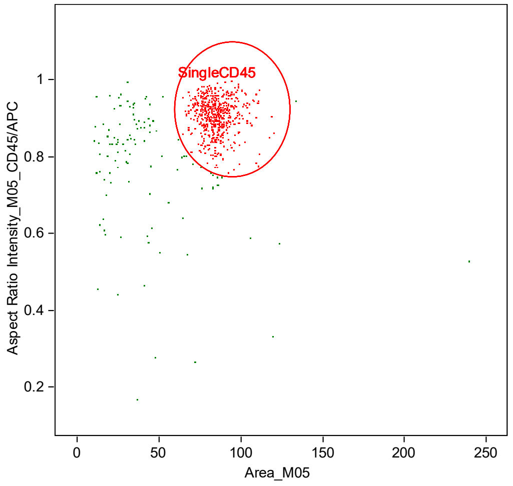

Step 3: Use a combination of the aspect ratio (between 0.75 to 1) and area (between 60-130 μm^2) to identify single CD45$^+$ cells in focus.

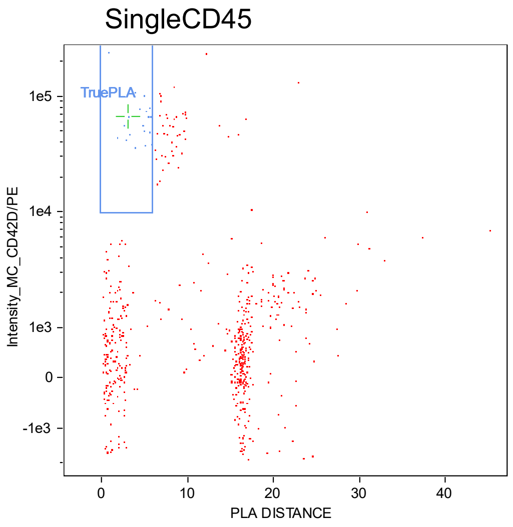

Step 4a: Identify PLT (CD42d; intensity > 10,000) that are in contact with WBC. Use a center-to-center cutoff distance of 6 μm to identify “true” platelet-leukocyte aggregates. Image #142252: The centers of the WBC (red) and PLT (yellow) are ~3 μm apart.

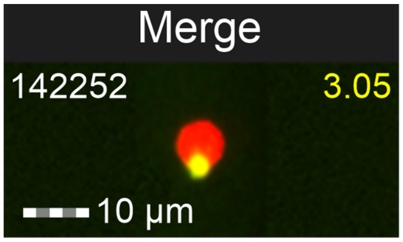

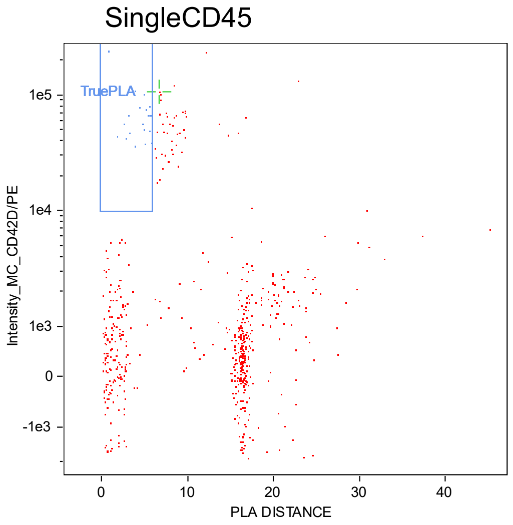

Step 4b: Identify PLT (CD42d; intensity > 10,000) that are in contact with WBC. Use a center-to-center cutoff distance of 6 μm to identify “true” platelet-leukocyte aggregates. Image #30724: The centers of the WBC (red) and PLT (yellow) are ~6.7 μm apart. Note the gap between the cells.

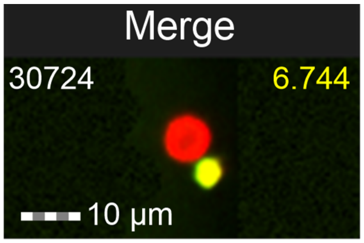

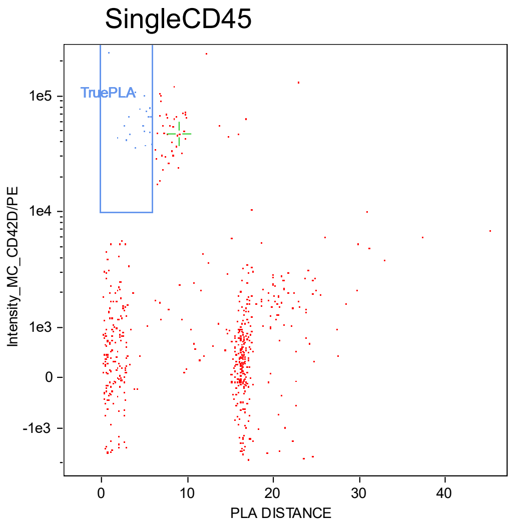

Step 4c: Identify PLT (CD42d; intensity > 10,000) that are in contact with WBC. Use a center-to-center cutoff distance of 6 μm to identify “true” platelet-leukocyte aggregates. Image #44195: The centers of the WBC (red) and PLT (yellow) are ~9 μm apart. Note the large gap between the cells.

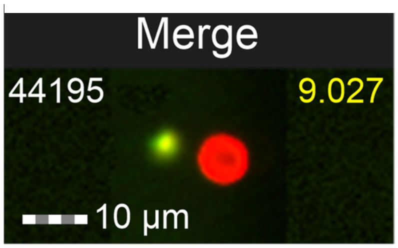

Supplementary Note: Calculating the Shear Rate and Shear Stress at particular points within a CIF Element

To calculate shear rate and shear stress experienced by cells flowing through channels with rectangular cross-section, the following equations may be used[1]:

$$\dot{\gamma_w} = \dot{\gamma_a} \times \left(\frac{2}{3}\right) \times \left(\frac{b^*}{f^*} + \frac{a^*}{f^*} \times \frac{1}{n}\right) \qquad Supplementary\ Equation\ (1)$$

$$\dot{\gamma_a} = \left(\frac{Q}{10 \times W \times H^2}\right) \times \left(1 + \frac{H}{W}\right) \times f^* \times \left(\frac{H}{W}\right) \qquad Supplementary\ Equation\ (2)$$

$$\tau_w = K[\dot{\gamma_w}]^n \qquad Supplementary\ Equation\ (3)$$

, where

$\dot{\gamma_w}$ = wall shear rate (s^{-1})

$\dot{\gamma_a}$ = apparent shear rate (s^{-1})

τ_w = wall shear stress (Pa)

$a^*, b^*, and\ f^*$ = geometric constants for a rectangular duct

n = power-law index (dimensionless)

K = consistency index (Pa.s^n)

Q = flow rate (μL min^{-1})

H, W = Dimensions of the rectangular cross-section at the point-of-interest; *W*>*H* (mm).

(Note: Supplementary Equation 2 has been slightly modified from the reference cited to output a value directly in s^{-1} units.) Constants *a**, *b**, and *f** are obtained from the literature[1,2], with respect to the height and width dimensions of the rectangular cross-section (*H*, *W*; *H*<*W*). While exact values for the power-law index (*n*) and consistency index (*K*) ideally should be determined empirically[1], we can obtain estimates for whole blood (WB) from the literature. Hussain *et al*[3] and Shibeshi and Collins[4] reported *n* and *K* values for WB to be 0.708 and 17 mPa.s^n, respectively, while Kim *et al*[5] determined these values to be 0.828 and 9.267 cP.s^{n-1} (9.267 mPa.s^n). Walburn and Schneck[6] presented a hematocrit (Hct)-based model for calculating *n* and *K*, using the equations $n = 1 - 0.00499 \times \mathrm{Hct}$ and $k = 0.0148e^{0.0512 \times Hct}$ to obtain these parameters. Since these blood-specific parameters can be difficult to quantify precisely, we used all of the values/equations above to calculate a range of shear rate and shear stress estimates experienced in our microfluidic devices, presented in Supplementary Table 3 for Designs 1 to 4.

Our calculations show that for our *in vitro* experiments, the average wall shear rate experienced in the ~20 μm wide gaps between filtration posts was 15.8 to 25.1 s^{-1}, with the corresponding shear stress ranging from 0.075 to 0.166 Pa (0.75 to 1.66 dynes cm^{-2}), depending on the device design, flowrate, and the pair of n and k values used. These shear rate and stress values are on the low end of the ranges for physiological shear rate (15-200 s^{-1}) and shear stress (0.5-7 dynes cm^{-2}) experienced *in vivo* by blood cells in veins, and significantly below the shear rate (300-800 s^{-1}) and shear stress (10-40 dynes cm^{-2}) typically experienced in large arteries[7,8]. Furthermore, these values are well below the shear rates and stresses typically associated with PLT activation[9,10].

The shear rates and stresses experienced at the start of each CIF element (see Supplementary Fig. 1), where these values would typically be the highest in the central flow path[11], were also calculated for our *in vitro* experiments. The wall shear rate ranged from 1675 to 3743 s^{-1}, while

the shear stress was between 3.4 and 8.2 Pa (34 to 82 dynes cm^{-2}), with estimates varying quite significantly depending on the model (and device design) used; these values are comparable to the physiological shear rate (450-2000 s^{-1}) and shear stress (16-95 dynes cm^{-2}) in micro-arterioles and capillaries[8,12]. These calculated values are also below the vWF unfolding thresholds (5000-5522 s^{-1}) and some of the higher shear rates/stresses associated with PLT activation that are reported in the literature[8,13]. Since the shear experienced within the CIF flow channels during our typical experimental protocol is not significantly different from physiological shear levels, no evidence of PLT or cell activation is observed – particularly in the filtration gaps, which impose relatively low shear conditions on the processed blood samples.

Supplementary Tables

	CIF (n=8)	Sham (n=8)	p-value
**Major Cell Types**			
**RBC Slope [95% CI]**	**-0.1008 [-0.1353 to -0.0664]**	**-0.0602 [-0.0742 to -0.0461]**	**0.0313**
PLT Slope [95% CI]	0.0013 [-0.0931 to 0.0958]	0.0034 [-0.0600 to 0.0668]	0.9713
**WBC Slope [95% CI]**	**-0.2919 [-0.3532 to -0.2306]**	**-0.0235 [-0.0808 to 0.0338]**	**<0.0001**
**Leukocyte Subtypes**			
**PMN Slope [95% CI]**	**0.3346 [0.1216 to 0.5475]**	**1.595 [1.358 to 1.831]**	**<0.0001**
**LYM Slope [95% CI]**	**-0.3935 [-0.4471 to -0.3398]**	**-0.3110 [-0.3475 to -0.2745]**	**0.0125**
**MON Slope [95% CI]**	**-0.1269 [-0.2385 to -0.01529]**	**0.1533 [0.04588 to 0.2607]**	**0.0004**
EOS Slope [95% CI]	-0.5962 [-0.8028 to -0.3896]	-0.3885 [-0.5142 to -0.2628]	0.09
BAS Slope [95% CI]	-0.2461 [-0.4120 to -0.08020]	-0.01156 [-0.2147 to 0.1915]	0.0776

Supplementary Table 1: Changes in percent baseline (CIF vs. sham). Simple linear regression analyses comparing changes in percent baseline of circulating cells over time in CIF and sham animals. P-values for differences between slopes in CIF and sham groups.

Parameter	Variable	Estimate	Standard Error	95% CI	p-value
**a: Final WBC Percent of Baseline**					
β0	Intercept	17.86	46.29	-85.29 to 121.0	0.7078
**β1**	**Group (CIF)**	**-94.83**	**28.79**	**-159.0 to -30.67**	**0.0081**
β2	Fluid Given	-0.1154	0.7223	-1.725 to 1.494	0.8763
β3	Fluid Removed	5.462	3.105	-1.455 to 12.38	0.1090
β4	Weight (g)	0.1747	0.09043	-0.02680 to 0.3762	0.0822
β5	Sex (Female)	-14.41	7.150	-30.34 to 1.522	0.0716
**b: Final RBC Percent of Baseline**					
**β0**	**Intercept**	**53.59**	**21.77**	**5.077 to 102.1**	**0.0336**
β1	Group (CIF)	-13.99	13.54	-44.17 to 16.19	0.3259
β2	Fluid Given	-0.6513	0.3397	-1.408 to 0.1056	0.0842
β3	Fluid Removed	1.520	1.460	-1.734 to 4.774	0.3224
β4	Weight (g)	0.07025	0.04253	-0.02451 to 0.1650	0.1296
β5	Sex (Female)	4.491	3.363	-3.001 to 11.98	0.2113
**c: Final PLT Percent of Baseline**					
β0	Intercept	-18.98	52.26	-135.4 to 97.47	0.7241
β1	Group (CIF)	-40.92	32.51	-113.4 to 31.52	0.2367
β2	Fluid Given	-0.9724	0.8155	-2.789 to 0.8446	0.2606
β3	Fluid Removed	5.198	3.505	-2.612 to 13.01	0.1689
**β4**	**Weight (g)**	**0.2455**	**0.1021**	**0.01804 to 0.4730**	**0.0370**
β5	Sex (Female)	15.85	8.072	-2.139 to 33.83	0.0780

Supplementary Table 2: Association of final percent of baseline with various factors. Multiple linear regression analyses comparing final (a) WBC, (b) RBC, and (c) PLT percents of baseline to experimental group (CIF), fluid given, fluid removed, animal weight, and animal sex (female). (a) Presence in the CIF group (as compared to sham) was independently associated with greater reductions in final WBC percent. (b) Presence in the CIF group was not independently associated with changes in final (b) RBC or (c) PLT percents of baseline. (c) Increasing weight was only independently associated with increasing final PLT percents of baseline.

Parameter	Design 1	Design 2	Design 3	Design 4
**a: Gaps between the pill-shaped posts**				
H/*W*	0.135	0.135	0.135	0.115
*a**	0.3875	0.3875	0.3875	0.4017
*b**	0.8857	0.8857	0.8857	0.9001
*f**	0.8488	0.8488	0.8488	0.8679
Flow rate (*Q*) (µL min^{-1})	0.068	0.086	0.086	0.086
Apparent shear rate ($\dot{\gamma}_a$) (s^{-1})	14.9	19.0	18.8	22.2
Wall shear rate ($\dot{\gamma}_w$) (s^{-1})	15.8 - 16.8	20.1. - 21.4	19.9 - 21.2	23.5 - 25.1
Wall shear stress (τ_w) (mPa)	75.3 - 125.3	92.3 - 148.6	91.6 - 147.6	105.5 - 166.3
**b: Start of the CIF element**				
H/*W*	0.822	0.763	0.632	0.590
*a**	0.2150	0.2172	0.2260	0.2306
*b**	0.6811	0.6858	0.7020	0.7093
*f**	0.5974	0.6019	0.6186	0.6265
Flow rate (*Q*) (µL min^{-1})	150	150	150	150
Apparent shear rate ($\dot{\gamma}_a$) (s^{-1})	3405.0	2857.6	1869.3	1602.2
Wall shear rate ($\dot{\gamma}_w$) (s^{-1})	3556.8 - 3742.5	2985.3 - 3141.6	1953.8 - 2057.3	1675.1 - 1764.5
Wall shear stress (τ_w) (mPa)	5757.7 - 8178.3	5086.6 - 7115.6	3769.3 - 5080.7	3381.1 - 4496.1

Supplementary Table 3: Shear in CIF devices. Calculated shear rate and shear stress results within CIF elements for *in vitro* experiments (a) in the filtration gaps between adjacent posts, and (b) at the start of the CIF element. *H*/*W* is the ratio of the smallest dimension to the largest dimension of the rectangular cross-section; *a**, *b**, and *f** are constants calculated based on *H*/*W* (see Supplementary Note above). In section (a), *Q* is the average flow rate through individual gaps, while in section (b), it is the input flow rate for each of the eight identical CIF elements which are arranged in parallel. Shear values are estimated based on equations in the literature for flow through rectangular channels and for the rheological properties of blood (see Supplementary Note above).

Supplementary Figures

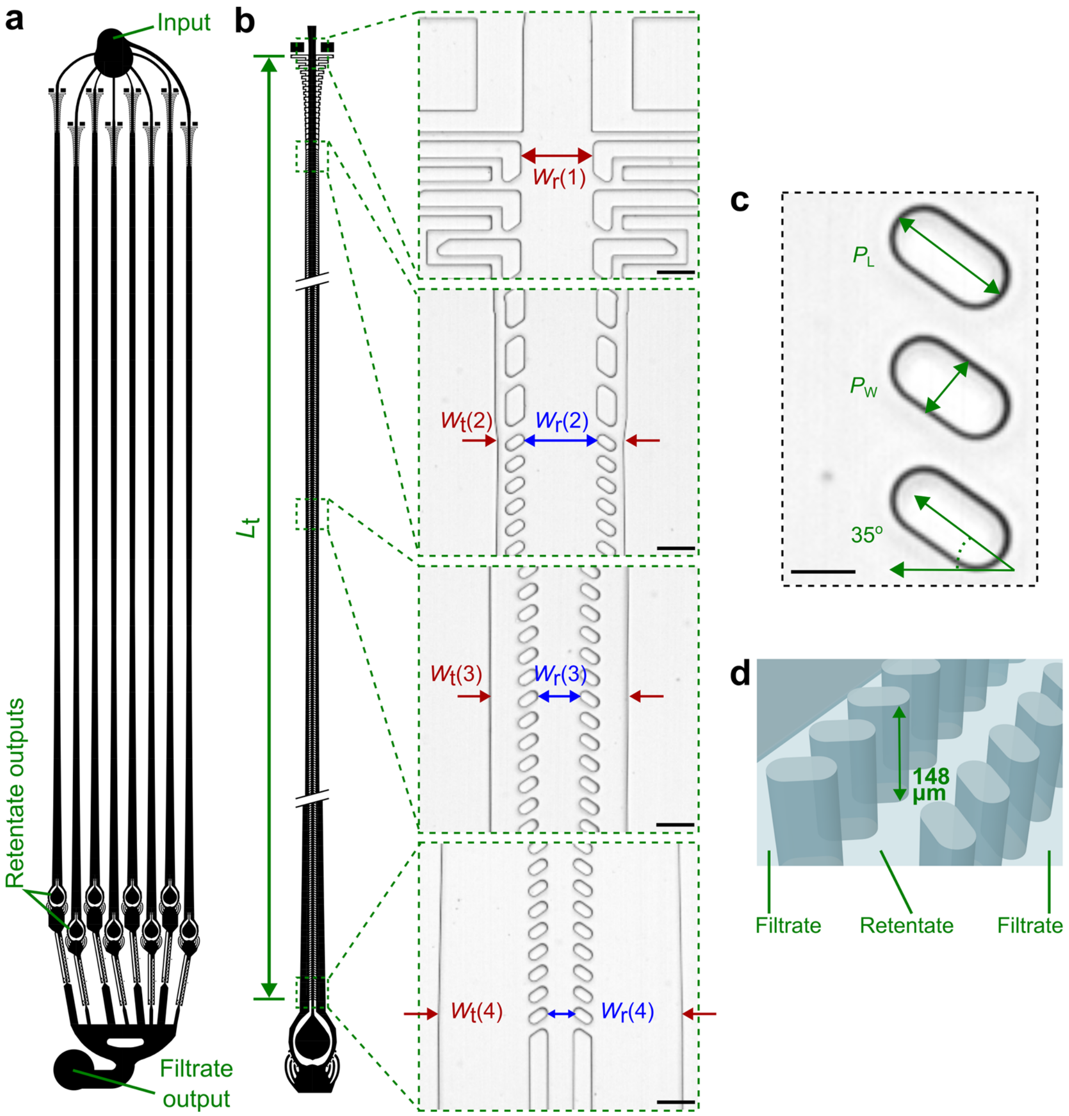

e

	f_{gap}^*	$W_r(1)$ (µm)	$W_r(2)$ (µm)	$W_t(2)$ (µm)	$W_r(3)$ (µm)	$W_t(3)$ (µm)	$W_r(4)$ (µm)	$W_t(4)$ (µm)	L_t (mm)	No. of filtration points	P_L (µm)	P_W (µm)
**Design 1**	0.72×10^{-3}	180	180	308.1	85.6	312.9	70	641.5	63.76	2156	56	28
**Design 2**	1.00×10^{-3}	194	194	322.1	94.5	331.4	70	640.1	49.97	1692	56	28
**Design 3**	1.28×10^{-3}	234	234	362.1	136.7	399.2	70	640.3	50.03	1702	56	28
**Design 4**	1.70×10^{-3}	251	251	400.4	159.9	474	67	704.4	49.99	1702	62	31

Supplementary Figure 1: Dimensions of the four different CIF designs used in the study. (a) The overall architecture of an 8-element device. Each design has the same basic layout, however the retentate and filtrate channel widths along the length of the element differ, dictated by its particular f_{gap}^* value. (b) Details of a single element. Key regions (start, middle, and end) are shown. L_t: Total length of the element measured from the start of the element to the end of the

array of posts. $W_r(1)$: width of the retentate channel at element start. $W_r(2)$: width of the retentate channel at the start of the array of posts. $W_t(2)$: total width of the CIF element at the start of the array of posts. $W_r(3)$: width of the retentate channel at the midway point of the array of posts. $W_t(3)$: total width of the CIF element at the midway point of the array of posts. $W_r(4)$: width of the retentate channel at element end. $W_t(4)$: total width of the CIF element at element end. Scale bars: 100 μm. (c) Dimensions of the pill-shaped posts. P_L: length of a post. P_W: width of a post. Each post is angled 35°. Scale bar: 25 μm. (d) The depth of each CIF element is ~148 μm. (e) Description of various parameters of the four CIF element designs.

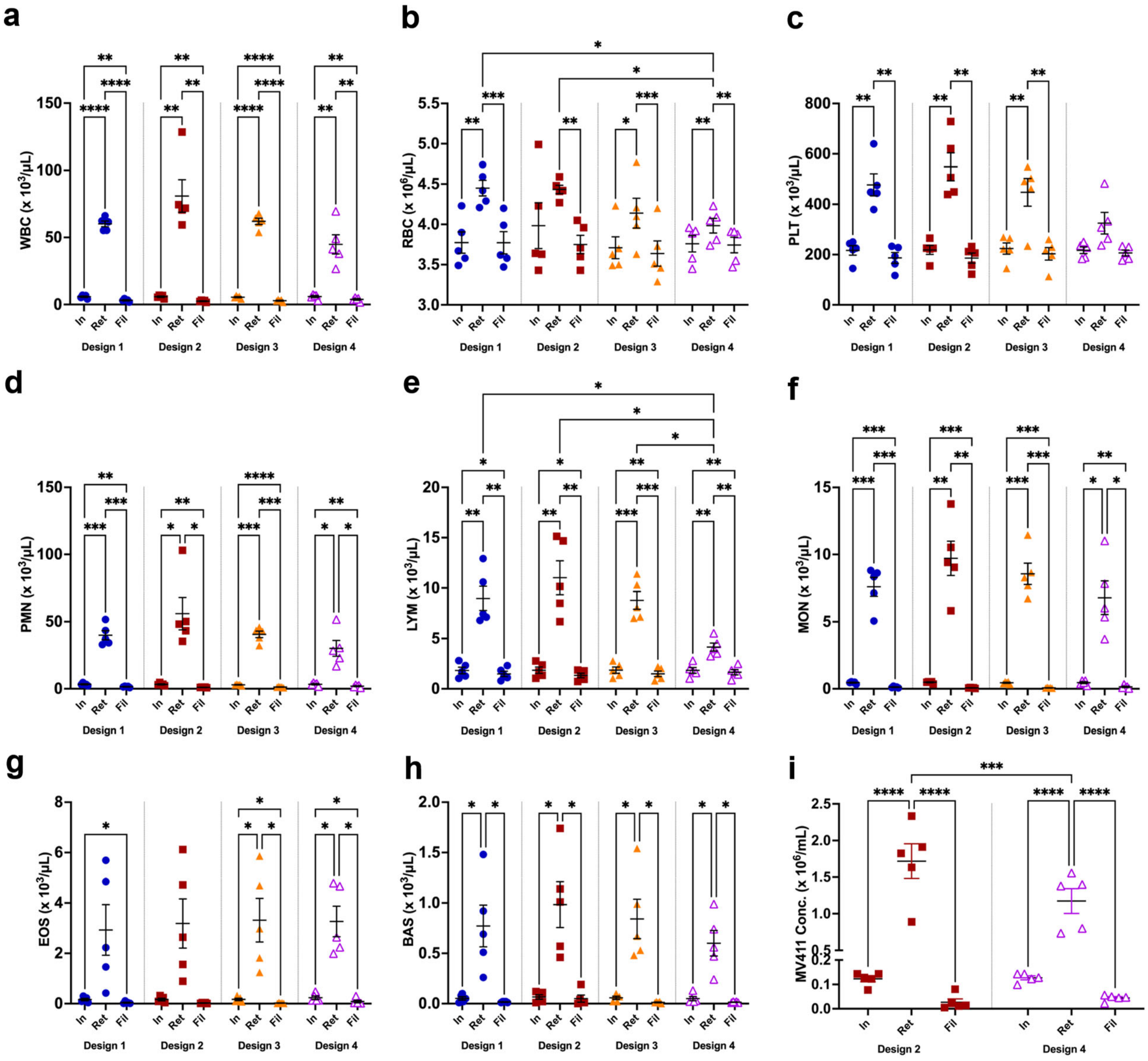

Supplementary Figure 2: Concentrations of cells in input, retentate, and filtrate across all designs. (a) All CIF designs had significantly higher WBC concentrations in the retentate and decreased WBC concentrations in the filtrate samples, as compared to the input sample. (b) Although RBC concentrations were higher in the retentate channel, their concentrations in the filtrate channel were similar to the input concentrations. (c) PLT concentrations were also higher in the retentate channel, but the filtrate concentrations were similar to input. (d-h) There were significant reductions in the three major WBC subsets (PMN, LYM, and MON) in the filtrate samples as compared to the input samples. (i) Only Designs 2 and 4 were run with MV-4-11 spiked blood samples. Both designs significantly increased the concentration of cancer cells in the retentate and significantly decreased it in the filtrate. In=Input, Ret=Retentate, Fil=Filtrate. Data shown as mean ± s.e.m. n=5. Data analyzed by 2-way RM ANOVA with Tukey's multiple comparison test. *p<0.05, **p<0.01, ***p<0.001, ****p<0.0001.

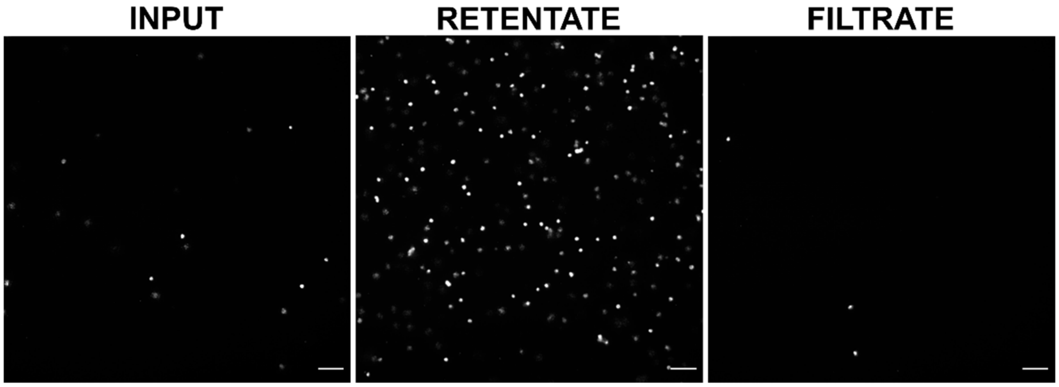

Supplementary Figure 3: Visualization of CIF separation performance with MV-4-11 cells. A visual comparison of the concentrations of MV-4-11 cells in input, retentate, and filtrate samples. All samples are undiluted and placed in a hemocytometer prior to fluorescent imaging. The concentration of MV-4-11 cells increases in the retentate and decreases in the filtrate significantly compared to the input. Scale bars: 100 µm.

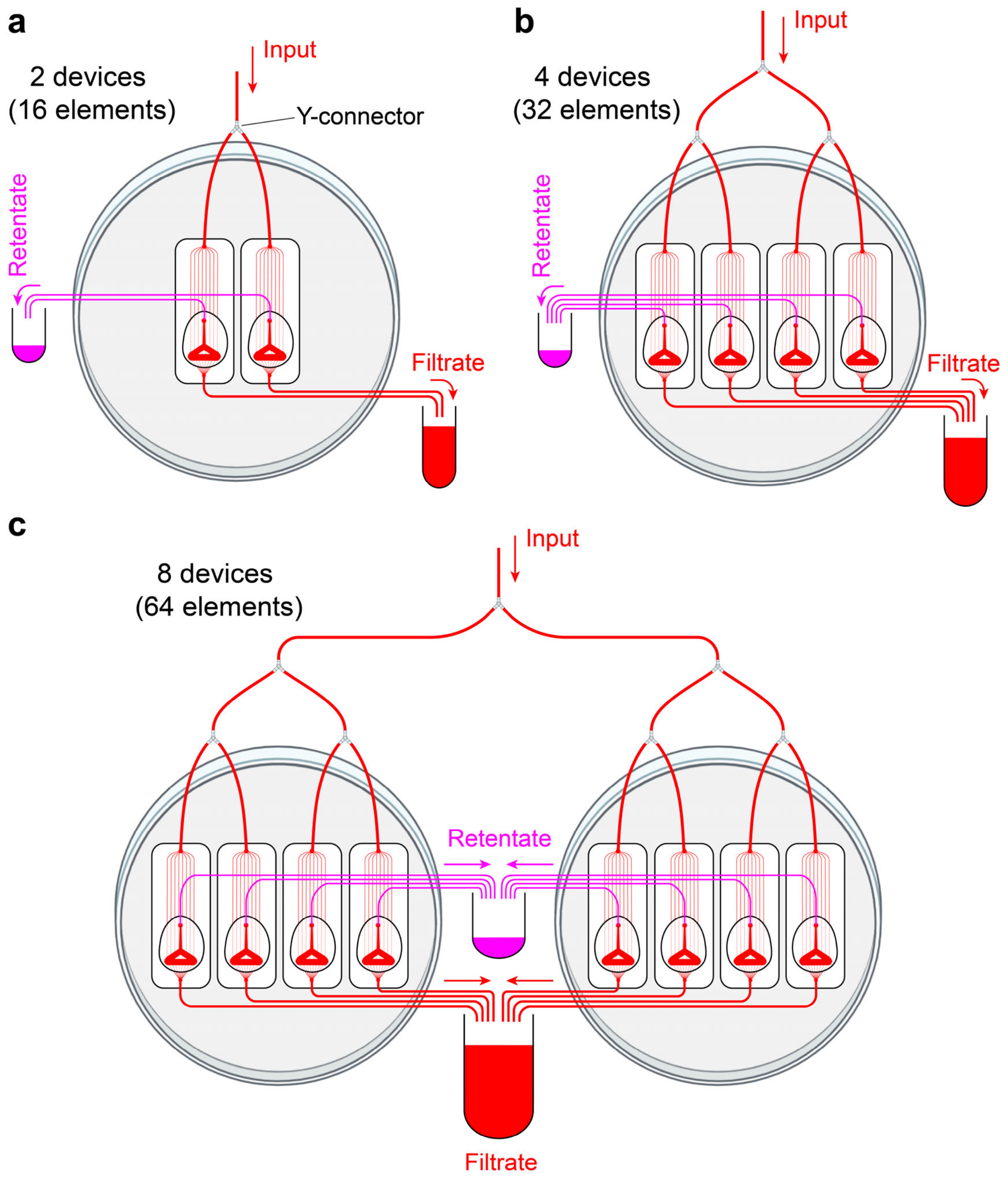

Supplementary Figure 4: Schematics of the experimental setups used for device multiplexing. Cell separation performance of one 8-element device was compared to that of two, four, and eight devices connected in parallel. (a) Setup using two 8-element devices. Input flow rate: 2.4 mL min^{-1}. Total internal volume (internal device volume plus input, retentate, and side tubing internal volumes): ~1.0 mL. (Partially created in BioRender. Iqbal, M. (2024) https://BioRender.com/i31i968) (b) Setup using four 8-element devices. Input flow rate: 4.8 mL min^{-1}. Total volume: ~2.1 mL. (Partially created in BioRender. Iqbal, M. (2024) https://BioRender.com/i31i968) (c) Setup using eight 8-element devices. Input flow rate: 9.6 mL min^{-1}. Total volume: ~4.2 mL. For each setup, input tubing is bifurcated using 1/16” Y-connectors. All retentate outputs are collected in one vial, and all filtrate outputs are collected in another. (Partically created in BioRender. Iqbal, M. (2024) https://BioRender.com/i31i968)

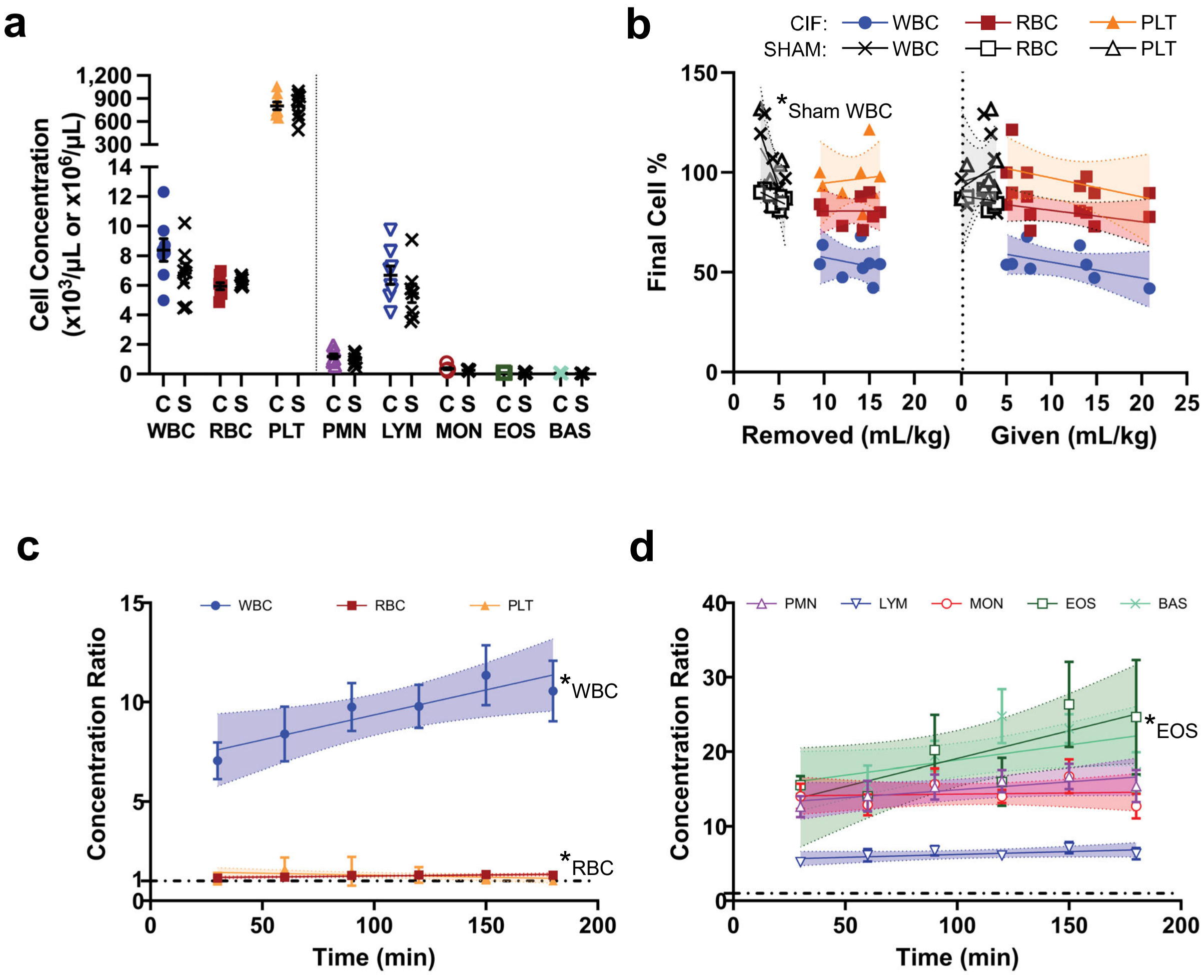

Supplementary Figure 5: Complete blood counts and trends in cell counts during CIF-based leukapheresis in rats. (a) Initial cell concentrations were similar between both the CIF (C) and sham (S) groups. (b) Univariate linear regression analysis showed no effect of fluid changes on changes in WBC (●), RBC (■), and PLT (▲) in the CIF group. In the sham group alone (×, □, and △ for WBC, RBC, and PLT, respectively) more fluid removal was associated with decreased final WBC percent of baseline (*p=0.0437). (c) During the leukapheresis procedure, the concentration ratios of WBC and, to a lesser extent, RBC continued to rise whereas the PLT concentration ratio remained flat. (d) The concentration ratios of the WBC subsets were mostly stable, with only the concentration ratio of EOS (□, *p=0.0458) increasing over time. The overall concentration ratios of the larger subsets (PMN △, MON ○, EOS, and BAS ×) were higher than the small subset of LYM (▽)throughout the leukapheresis procedure, similar to the overall concentration ratios as seen in Figure 4j. Data shown as mean ± SEM and linear regression best-fit lines with 95% confidence interval bands. *p<0.05 for non-zero slopes using linear regression analysis. n=8 for both CIF and sham groups.

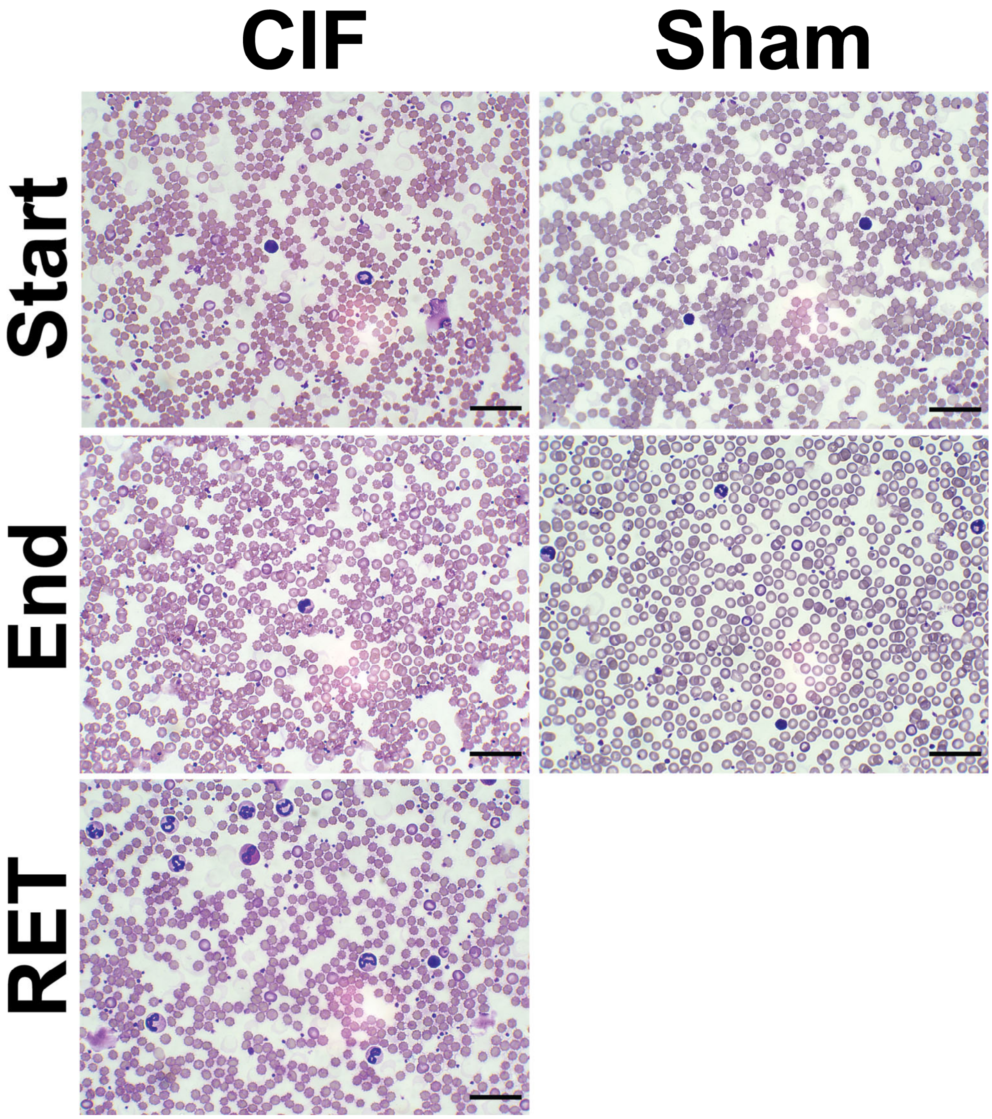

Supplementary Figure 6: Wright stains of peripheral rat blood from animals in the CIF and Sham groups. There is no appreciable schistocytosis seen in either group, nor at the beginning or end of the procedures. The RET image is blood from the retentate in the CIF group. Scale bars: 30 µm.

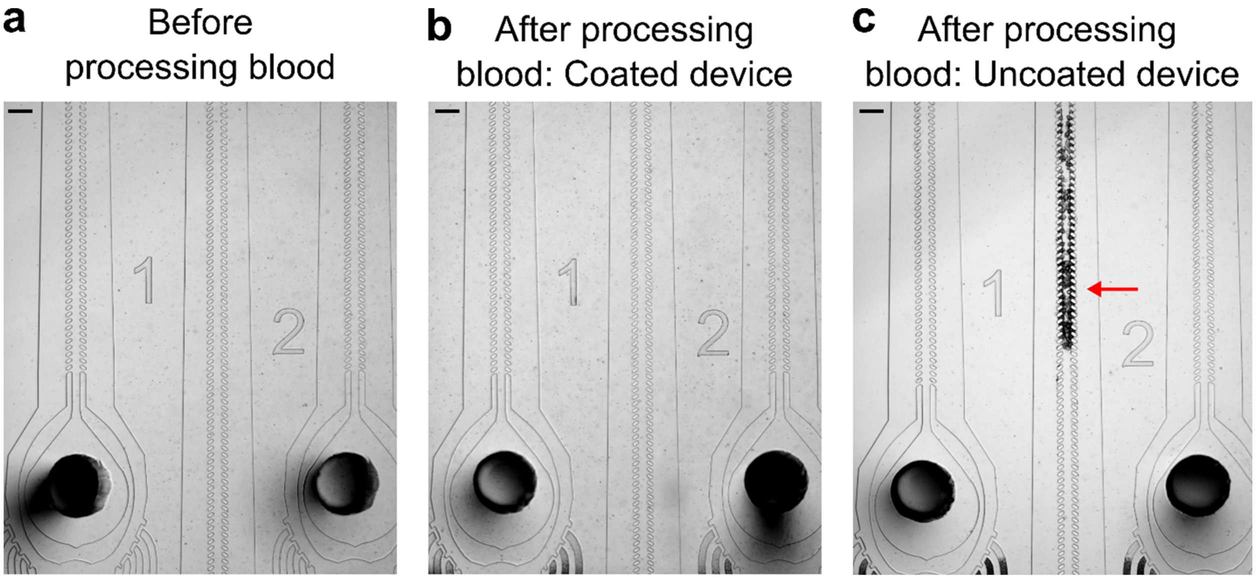

Supplementary Figure 7: Comparison of a coated device with an uncoated device. Photographs of 8-element CIF devices (a) before processing a blood sample and (b, c) after perfusion with undiluted whole blood at 1.2 mL min^{-1} followed by flushing with phosphate-buffered saline (PBS). (b) The coated device was treated with an aqueous 1% mPEG-Silane (MW 5000 Da) solution, followed by flushing and overnight incubation with 1% BSA in PBS. (c) The uncoated device was incubated with PBS only. The channels and posts in the coated device are observed to be clean and unclogged, whereas the uncoated device has a significant buildup of clumps/cellular material (indicated by the red arrow), likely due to adhesion to the posts resulting from lack of surface treatment. The middle element (element no. 2) in panel (c) contains most of the clumps pictured, but individual cells can be seen sticking to the posts in the adjacent elements (no. 1 & 3) as well. Scale bars: 200 μm.

Supplementary References

1 Son, Y. Determination of shear viscosity and shear rate from pressure drop and flow rate relationship in a rectangular channel. *Polymer* **48**, 632-637 (2007). https://doi.org:https://doi.org/10.1016/j.polymer.2006.11.048

2 Hartnett, J. P. & Kostic, M. Heat Transfer to Newtonian and Non-Newtonian Fluids in Rectangular Ducts. *Advances in Heat Transfer* **19**, 247-356 (1989). https://doi.org:https://doi.org/10.1016/S0065-2717(08)70214-4

3 Hussain, M. A., Kar, S. & Puniyani, R. R. Relationship between power law coefficients and major blood constituents affecting the whole blood viscosity. *Journal of Biosciences* **29**, 329-337 (1999). https://doi.org:https://doi.org/10.1007/BF02941247

4 Shibeshi, S. S. & Collins, W. E. The Rheology of Blood Flow in a Branched Arterial System. *Appl Rheol* **15**, 398-405 (2005). https://doi.org:10.1901/jaba.2005.15-398

5 Kim, S., Cho, Y. I., Jeon, A. H., Hogenauer, B. & Kensey, K. R. A new method for blood viscosity measurement. *Journal of Non-Newtonian Fluid Mechanics* **94**, 46-56 (2000). https://doi.org:https://doi.org/10.1016/S0377-0257(00)00127-0

6 Walburn, F. J. & Schneck, D. J. A constitutive equation for whole human blood. *Biorheology* **13**, 201-210 (1976). https://doi.org:10.3233/bir-1976-13307

7 Rana, A., Westein, E., Niego, B. & Hagemeyer, C. E. Shear-Dependent Platelet Aggregation: Mechanisms and Therapeutic Opportunities. *Front Cardiovasc Med* **6**, 141 (2019). https://doi.org:10.3389/fcvm.2019.00141

8 Chan, C. H. H. *et al.* Shear-dependent platelet aggregation size. *Artif Organs* **44**, 1286-1295 (2020). https://doi.org:10.1111/aor.13783

9 Holme, P. A. *et al.* Shear-induced platelet activation and platelet microparticle formation at blood flow conditions as in arteries with a severe stenosis. *Arterioscler Thromb Vasc Biol* **17**, 646-653 (1997). https://doi.org:10.1161/01.atv.17.4.646

10 Nobili, M., Sheriff, J., Morbiducci, U., Redaelli, A. & Bluestein, D. Platelet activation due to hemodynamic shear stresses: damage accumulation model and comparison to in vitro measurements. *ASAIO J* **54**, 64-72 (2008). https://doi.org:10.1097/MAT.0b013e31815d6898

11 Xia, H., Strachan, B. C., Gifford, S. C. & Shevkoplyas, S. S. A high-throughput microfluidic approach for 1000-fold leukocyte reduction of platelet-rich plasma. *Sci Rep* **6**, 35943 (2016). https://doi.org:10.1038/srep35943

12 Koutsiaris, A. G. *et al.* Volume flow and wall shear stress quantification in the human conjunctival capillaries and post-capillary venules in vivo. *Biorheology* **44**, 375-386 (2007).

13 Han, D., Zhang, J., Griffith, B. P. & Wu, Z. J. Models of Shear-Induced Platelet Activation and Numerical Implementation With Computational Fluid Dynamics Approaches. *J Biomech Eng* **144** (2022). https://doi.org:10.1115/1.4052460
