# Supplementary material for: Ultra-low extracorporeal volume microfluidic leukapheresis is safe and effective in a rat model
Source: Nat Commun. 2025 Feb 24;16:1930. doi: 10.1038/s41467-025-57003-5 (PMC11850925; doi:10.1038/s41467-025-57003-5)
Supplement: Supplementary file 2 — Description of Additional Supplementary Files [file 41467_2025_57003_MOESM2_ESM.pdf]

Description of Additional Supplementary Files

Supplementary Movie 1. Removal of MV-4-11 cells from whole blood. A bright field + fluorescent view of the separation of the MV-4-11 cancer cells in a CIF element. MV-4-11 cells stained with DiI dye are spiked at ~10^5 per mL in whole blood. The sample is flowing at 1.2 mL min^{-1} through 8 elements in parallel. As can be seen in the video, most MV-4-11 cells (appearing as bright spots) stay in the retentate channel.

Supplementary Movie 2. Processing undiluted whole blood (WB) with a CIF device. A bright field view of the overall process of running WB through an 8-element CIF (Design 2) device. WB is run through the device at 1.2 mL min^{-1} using a syringe pump. After the sample is processed, PBS is perfused at the same flow rate to demonstrate the clog-free operation of the device.
