# Supplementary material for: Ultra-low extracorporeal volume microfluidic leukapheresis is safe and effective in a rat model
Source: Nat Commun. 2025 Feb 24;16:1930. doi: 10.1038/s41467-025-57003-5 (PMC11850925; doi:10.1038/s41467-025-57003-5)
Supplement: Supplementary file 5 — Reporting Summary [file 41467_2025_57003_MOESM5_ESM.pdf]

nature portfolio

Corresponding author(s): Fong Lam and Sergey Shevkoplyas

Last updated by author(s): 12/30/2024

Reporting Summary

Nature Portfolio wishes to improve the reproducibility of the work that we publish. This form provides structure for consistency and transparency in reporting. For further information on Nature Portfolio policies, see our Editorial Policies and the Editorial Policy Checklist.

Statistics

For all statistical analyses, confirm that the following items are present in the figure legend, table legend, main text, or Methods section.

n/a	Confirmed	
☐	☒	The exact sample size (*n*) for each experimental group/condition, given as a discrete number and unit of measurement
☐	☒	A statement on whether measurements were taken from distinct samples or whether the same sample was measured repeatedly
☐	☒	The statistical test(s) used AND whether they are one- or two-sided<br>*Only common tests should be described solely by name; describe more complex techniques in the Methods section.*
☐	☒	A description of all covariates tested
☐	☒	A description of any assumptions or corrections, such as tests of normality and adjustment for multiple comparisons
☐	☒	A full description of the statistical parameters including central tendency (e.g. means) or other basic estimates (e.g. regression coefficient) AND variation (e.g. standard deviation) or associated estimates of uncertainty (e.g. confidence intervals)
☐	☒	For null hypothesis testing, the test statistic (e.g. *F*, *t*, *r*) with confidence intervals, effect sizes, degrees of freedom and *P* value noted<br>*Give P values as exact values whenever suitable.*
☒	☐	For Bayesian analysis, information on the choice of priors and Markov chain Monte Carlo settings
☒	☐	For hierarchical and complex designs, identification of the appropriate level for tests and full reporting of outcomes
☒	☐	Estimates of effect sizes (e.g. Cohen's *d*, Pearson's *r*), indicating how they were calculated

Our web collection on statistics for biologists contains articles on many of the points above.

Software and code

Policy information about availability of computer code

Data collection	Data collection from laboratory equipment was from the manufacturer's software (adinstrument LabChart 8; Heska ePOC; Heska HT5; Luminex IDEAS 6.2, Sysmex XS-1000i). Data collected by outsourced companies (Eve Technologies) were done per their practices. Microsoft Excel was used to organize data in which there were no automated systems for export (e.g., H&E analyses, hemocytometer counting of cells). No custom software was used.
Data analysis	Microsoft Excel was used to organize CSV data exported from equipment. GraphPad Prism 10 was used to analyze the data.

For manuscripts utilizing custom algorithms or software that are central to the research but not yet described in published literature, software must be made available to editors and reviewers. We strongly encourage code deposition in a community repository (e.g. GitHub). See the Nature Portfolio guidelines for submitting code & software for further information.

Data

Policy information about availability of data

All manuscripts must include a data availability statement. This statement should provide the following information, where applicable:

- Accession codes, unique identifiers, or web links for publicly available datasets
- A description of any restrictions on data availability
- For clinical datasets or third party data, please ensure that the statement adheres to our policy

All data supporting the findings of this study are available within the paper and its Supplementary Information.

Research involving human participants, their data, or biological material

Policy information about studies with human participants or human data. See also policy information about sex, gender (identity/presentation), and sexual orientation and race, ethnicity and racism.

Reporting on sex and gender	For the retrospective cohort study, we reported gender based on what was listed in the subjects' chart. For in vitro experiments with donated human whole blood, we were blinded to the gender of the donors.
Reporting on race, ethnicity, or other socially relevant groupings	For the retrospective cohort study, we reported race and ethnicity based on what was listed in the subjects' chart as a descriptor of the patient population that our quaternary center served. For in vitro experiments with donated human whole blood, we were blinded to the race or ethnicity of the donors.
Population characteristics	Part of our study included a retrospective cohort study to describe the field of pediatric leukapheresis as done clinically in a large quaternary center. In vitro studies used whole blood from a regional blood donation center and we were blinded to the population characteristics.
Recruitment	We performed a retrospective cohort study (2011-2022), so did not prospectively recruit subjects.
Ethics oversight	Human subjects research was approved through the Institutional Review Board at Baylor College of Medicine (H-3342) and the University of Houston (16272-01).

Note that full information on the approval of the study protocol must also be provided in the manuscript.

Field-specific reporting

Please select the one below that is the best fit for your research. If you are not sure, read the appropriate sections before making your selection.

☒ Life sciences ☐ Behavioural & social sciences ☐ Ecological, evolutionary & environmental sciences

For a reference copy of the document with all sections, see nature.com/documents/nr-reporting-summary-flat.pdf

Life sciences study design

All studies must disclose on these points even when the disclosure is negative.

Sample size	For the retrospective cohort study, we used a convenience sample size to describe the field of pediatric leukapheresis as done clinically in a large quaternary center. For the in vitro studies, we estimated that we would need 4 runs per group to identify a 50% decrease in WBC with an SD 20% in each group using a 1:1 allocation. For the in vivo studies, we estimated that we would need 4 animals in the CIF group and 4 animals in the sham group to identify a 50% decrease in WBC with an SD 20% in both groups using a 1:1 allocation. Because we took sex as a biologic variable, we had 4 male and 4 female rats per group.
Data exclusions	No data were excluded from the studies. There were some missing data due to either lack of sample, non-documentation in the medical record, or inability to compute (e.g., could not calculate a change in RBC counts if there were only one RBC concentration measured within our a priori window.
Replication	Replication/reproducibility was verified by performing experiments using different devices on different days with different blood units. Furthermore, we performed in vitro studies in a repeated measures fashion (same donor unit split among different device designs and among multiplexed (1-, 2-. 4, and 8-CIF designs).
Randomization	Randomization was not relevant to the study because we were comparing different CIF designs using the same blood units. In rat experiments, we performed extensive multiple variable linear regression analysis to account for sex, fluid given, fluid removed, weight, and device (CIF vs sham).
Blinding	Blinding was not performed because investigators could easily see which arms were being run (either device design, multiplexing, and rat CIF vs Sham).

Reporting for specific materials, systems and methods

We require information from authors about some types of materials, experimental systems and methods used in many studies. Here, indicate whether each material, system or method listed is relevant to your study. If you are not sure if a list item applies to your research, read the appropriate section before selecting a response.

Materials & experimental systems

n/a	Involved in the study
☐	☒ Antibodies
☐	☒ Eukaryotic cell lines
☒	☐ Palaeontology and archaeology
☐	☒ Animals and other organisms
☐	☒ Clinical data
☒	☐ Dual use research of concern
☒	☐ Plants

Methods

n/a	Involved in the study
☒	☐ ChIP-seq
☐	☒ Flow cytometry
☒	☐ MRI-based neuroimaging

Antibodies

Antibodies used

WB (100 µL) was labeled with allophycocyanin-conjugated mouse anti-rat CD45 (0.5 µg/test; clone OX-1; Invitrogen), phycoerythrin-conjugated Armenian hamster anti-mouse/rat CD42d (1 µg/test; clone 1C2; Invitrogen), and fluorescein isothiocyanate-conjugated Armenian hamster anti-mouse/rat CD61 (1 µg/test; clone2C9.G3; Invitrogen) antibodies for 20 minutes at room temperature. Non-immune IgG control antibodies were used to assess for non-specific binding.

Validation

WB (100 µL) was labeled with allophycocyanin-conjugated mouse anti-rat CD45 (0.5 µg/test; clone OX-1; Invitrogen), phycoerythrin-conjugated Armenian hamster anti-mouse/rat CD42d (1 µg/test; clone 1C2; Invitrogen), and fluorescein isothiocyanate-conjugated Armenian hamster anti-mouse/rat CD61 (1 µg/test; clone2C9.G3; Invitrogen) antibodies for 20 minutes at room temperature. Non-immune IgG control antibodies were used to assess for non-specific binding.

Validation was per the manufacturer's website (www.thermofisher.com/antibody/)

Eukaryotic cell lines

Policy information about cell lines and Sex and Gender in Research

Cell line source(s)

Cell lines were originally obtained from ATCC. Per the manufacturer's website: MV-4-11 cells are macrophages that were isolated from the blast cells of a 10-year-old male with biphenotypic B-myelomonocytic leukemia and deposited by the Wistar Institute.

Authentication

Cells were authenticated by short terminal repeat (STR) profiling

Mycoplasma contamination

Cell lines tested negative for mycoplasma.

Commonly misidentified lines
(See ICLAC register)

None identified at ICLAC

Animals and other research organisms

Policy information about studies involving animals; ARRIVE guidelines recommended for reporting animal research, and Sex and Gender in Research

Laboratory animals

Male and female Sprague-Dawley outbred rats (Crl:CD(SD); Charles River, Wilmington, MA), ages 10-16 weeks, were used in this study.

Wild animals

None

Reporting on sex

Male and female Sprague-Dawley outbred rats (Crl:CD(SD); Charles River, Wilmington, MA), ages 10-16 weeks, were used in this study. We performed multiple variable linear regression analysis, with sex as one of the listed variables.

Field-collected samples

None

Ethics oversight

Animal subjects research was approved through the Institutional Animal Care and Use Committees at Baylor College of Medicine (AN-8224) and Michael E. DeBakey Veterans Affairs Medical Center (20K05.A).

Note that full information on the approval of the study protocol must also be provided in the manuscript.

Clinical data

Policy information about clinical studies
All manuscripts should comply with the ICMJEguidelines for publication of clinical research and a completedCONSORT checklist must be included with all submissions.

Clinical trial registration

N/A

Study protocol	N/A. (This was not a clinical trial. We performed a retrospective cohort study.)
Data collection	To understand the current workflow and WBC removal efficiency of centrifugal leukapheresis for hyperleukocytosis in leukemic children, we assessed 43 pediatric leukapheresis procedures performed in 37 patients from 2/2011 to 12/2022 in a large, urban, quaternary pediatric hospital.
Outcomes	This portion of the study was to descriptive the clinical practice of pediatric leukapheresis and identify areas for improvement (e.g., ECV, hemodynamic changes, time to pheresis).

Plants

Seed stocks	N/A
Novel plant genotypes	N/A
Authentication	N/A

Flow Cytometry

Plots

Confirm that:

☒ The axis labels state the marker and fluorochrome used (e.g. CD4-FITC).

☒ The axis scales are clearly visible. Include numbers along axes only for bottom left plot of group (a 'group' is an analysis of identical markers).

☒ All plots are contour plots with outliers or pseudocolor plots.

☒ A numerical value for number of cells or percentage (with statistics) is provided.

Methodology

Sample preparation	WB (100 µL) was labeled with allophycocyanin-conjugated mouse anti-rat CD45 (0.5 µg/test; clone OX-1; Invitrogen), phycoerythrin-conjugated Armenian hamster anti-mouse/rat CD42d (1 µg/test; clone 1C2; Invitrogen), and fluorescein isothiocyanate-conjugated Armenian hamster anti-mouse/rat CD61 (1 µg/test; clone2C9.G3; Invitrogen) antibodies for 20 minutes at room temperature. Non-immune IgG control antibodies were used to assess for non-specific binding. Samples were then fixed with 2% paraformaldehyde and placed at 4°C until analysis within 24 hours. Data were analyzed in IDEAS 6.2 (Luminex Corporation) software. Compensated flow cytometry files were run through the IDEAS 6.2 co-localization wizard. PLT-WBC aggregates were defined as the percentage of WBC that had PLT within 6 µm of each other's center (see example shown in Supplementary Methods).
Instrument	Amnis Imagestreamx Mk II, Luminex Corporation, Austin TX
Software	IDEAS 6.2, Luminex Corporation
Cell population abundance	Stated in the Supplementary Methods (with example images):<br>Step 1: Identify cells in focus by using the brightfield channel (CH01; 40X magnification). Cells in the most focus have a high Gradient RMS (65 to 100).<br>Step 2: Use the RMS population to identify CD45+ (WBC; intensity>15,000) cells.<br>Step 3: Use a combination of the aspect ratio (between 0.75 to 1) and area (between 60-130 µm2) to identify single CD45+ cells in focus.<br>Step 4a: Identify PLT (CD42d; intensity > 10,000) that are in contact with WBC. Use a center-to-center cutoff distance of 6 µm to identify "true" platelet-leukocyte aggregates. Image #142252: The centers of the WBC (red) and PLT (yellow) are ~3 µm apart.<br>Step 4b: Identify PLT (CD42d; intensity > 10,000) that are in contact with WBC. Use a center-to-center cutoff distance of 6 µm to identify "true" platelet-leukocyte aggregates. Image #30724: The centers of the WBC (red) and PLT (yellow) are ~6.7 µm apart. Note the gap between the cells.<br>Step 4c: Identify PLT (CD42d; intensity > 10,000) that are in contact with WBC. Use a center-to-center cutoff distance of 6 µm to identify "true" platelet-leukocyte aggregates. Image #44195: The centers of the WBC (red) and PLT (yellow) are ~9 µm apart. Note the large gap between the cells.
Gating strategy	Stated in the Supplementary Methods (with example images):<br>Step 1: Identify cells in focus by using the brightfield channel (CH01; 40X magnification). Cells in the most focus have a high Gradient RMS (65 to 100).

Step 2: Use the RMS population to identify CD45+ (WBC; intensity>15,000) cells.
Step 3: Use a combination of the aspect ratio (between 0.75 to 1) and area (between 60-130 µm2) to identify single CD45+ cells in focus.
Step 4a: Identify PLT (CD42d; intensity > 10,000) that are in contact with WBC. Use a center-to-center cutoff distance of 6 µm to identify "true" platelet-leukocyte aggregates. Image #142252: The centers of the WBC (red) and PLT (yellow) are ~3 µm apart.
Step 4b: Identify PLT (CD42d; intensity > 10,000) that are in contact with WBC. Use a center-to-center cutoff distance of 6 µm to identify "true" platelet-leukocyte aggregates. Image #30724: The centers of the WBC (red) and PLT (yellow) are ~6.7 µm apart. Note the gap between the cells.
Step 4c: Identify PLT (CD42d; intensity > 10,000) that are in contact with WBC. Use a center-to-center cutoff distance of 6 µm to identify "true" platelet-leukocyte aggregates. Image #44195: The centers of the WBC (red) and PLT (yellow) are ~9 µm apart. Note the large gap between the cells.

☒ Tick this box to confirm that a figure exemplifying the gating strategy is provided in the Supplementary Information.
